# Albumin Binder–Conjugated Fibroblast Activation Protein Inhibitor Radiopharmaceuticals for Cancer Therapy

**DOI:** 10.2967/jnumed.121.262533

**Published:** 2022-06

**Authors:** Mengxin Xu, Pu Zhang, Jie Ding, Junyi Chen, Li Huo, Zhibo Liu

**Affiliations:** 1 Radiochemistry and Radiation Chemistry Key Laboratory of Fundamental Science, Beijing National Laboratory for Molecular Sciences, College of Chemistry and Molecular Engineering, Peking University, Beijing, China;; 2 Department of Nuclear Medicine, Peking Union Medical College Hospital, Chinese Academy of Medical Science and Peking Union Medical College, Beijing, China; and; 3 Peking University–Tsinghua University Center for Life Sciences, Beijing, China

**Keywords:** albumin binder, FAP inhibitor, radionuclide therapy

## Abstract

Fibroblast activation protein (FAP) has become an attractive target for diagnosis and therapy, and a series of FAP inhibitor (FAPI)–based radiotracers has been developed and had excellent performance for diagnosis outcomes in clinical applications. Yet, their fast clearance and insufficient tumor retention have hampered their further clinical application in cancer treatment. In this study, we developed 2 albumin binder–conjugated FAPI radiotracers, TEFAPI-06 and TEFAPI-07. They were derived from FAPI-04 and were optimized by conjugating 2 types of well-studied albumin binders, 4-(*p*-iodophenyl) butyric acid moiety (TEFAPI-06) and truncated Evans blue moiety (TEFAPI-07), to try to overcome the above limitations at the expense of prolonging the blood circulation. **Methods:** TEFAPI-06 and TEFAPI-07 were synthesized and labeled with ^68^Ga, ^86^Y, and ^177^Lu successfully. A series of cell assays was performed to identify the binding affinity and FAP specificity in vitro. PET imaging, SPECT imaging, and biodistribution studies were performed to evaluate the pharmacokinetics in pancreatic cancer patient–derived xenograft (PDX) animal models. The cancer treatment efficacy of ^177^Lu-TEFAPI-06 and ^177^Lu-TEFAPI-07 were evaluated in pancreatic cancer PDX–bearing mice. **Results:** The binding affinities (dissociation constants) to FAP of ^68^Ga-TEFAPI-06 and ^68^Ga-TEFAPI-07 were 10.16 ± 2.56 nM and 7.81 ± 2.28 nM, respectively, which were comparable with that of ^68^Ga-FAPI-04. Comparative PET imaging of HT-1080-FAP and HT-1080 tumor–bearing mice and a blocking study showed the FAP-targeting ability in vivo of these 2 tracers. Compared with ^177^Lu-FAPI-04, PET imaging, SPECT imaging, and biodistribution studies of TEFAPI-06 and TEFAPI-07 demonstrated their remarkably enhanced tumor accumulation and retention, respectively. Notable tumor growth inhibition by ^177^Lu-TEFAPI-06 and ^177^Lu-TEFAPI-07 were observed, whereas the control group and the group treated by ^177^Lu-FAPI-04 showed a slight therapeutic effect. **Conclusion:** Two albumin binder–conjugated FAPI radiopharmaceuticals have been developed and evaluated in vitro and in vivo. Significantly improved tumor uptake and retention were observed, compared with the original FAPI tracer. Both ^177^Lu-TEFAPI-06 and ^177^Lu-TEFAPI-07 showed remarkable growth inhibition of PDX tumors, whereas the side effects were almost negligible, demonstrating that these radiopharmaceuticals are promising for further clinical translational studies.

## Visual Abstract

**Figure:**
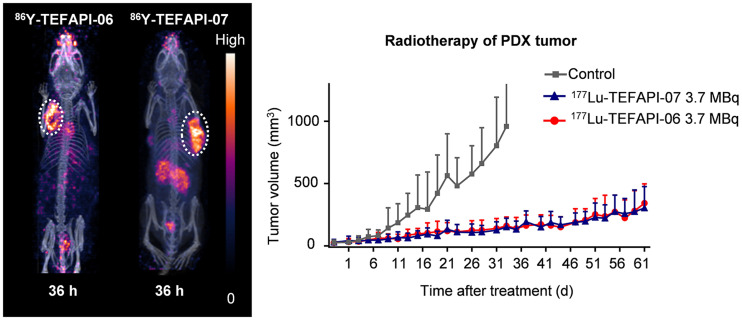

Fibroblast activation protein (FAP) is overexpressed in cancer-associated fibroblasts, which are one of the main tumor stroma components and constitute a major proportion of cells within the tumor (*1**,**2*). Though stromal cells are not malignant, the growth factor and chemokine produced by stromal cells, especially cancer-associated fibroblasts, can lead to the direct stimulation of tumor cell growth, migration, and progression (*3*). Considering the vital role in tumor survival and cancer growth, cancer-associated fibroblast–targeted diagnosis and therapy via the biomarker FAP have become an attractive strategy for tumor treatment (*4**,**5*). FAP-targeted radiopharmaceutical therapy might deliver therapeutic radioisotopes to cancer-associated fibroblasts. It damages the stromal cells and the neighboring tumor cells through the crossfire effect of the β- or α-emitting radionuclides, potentially augmenting the therapeutic efficacy (*6*).

Recently, a variety of quinolone-based FAP inhibitor (FAPI) radiopharmaceuticals has been developed and demonstrated excellent uptake in different FAP-positive tumors of cancer patients (*7*–*9*). For FAP-targeted radiotherapy, an emerging strategy is to directly modify the inhibitor structure to enhance tumor uptake and retention while keeping the accumulation in nontarget tissues unchanged or decreasing it (*10*–*12*). A series of FAPI probes, including FAPI-04, FAPI-21, and FAPI-46, has been successfully developed; their improved pharmacokinetic properties make them promising candidates for therapeutic outcome improvement (*13*–*16*). Though this is one of the optimal ways to develop therapeutic radiopharmaceuticals, the relatively rapid washout from the tumor is still a considerable limitation. Besides, it may be challenging to achieve notable enhancement of pharmacokinetic properties by subtle structure modification.

Several studies have shown that prolonging the blood circulation of drug molecules using albumin-binder moieties could remarkably improve the therapeutic dose (*17*). 4-(*p*-iodophenyl) butyric acid and truncated Evans blue moieties are the most widely used albumin binders. The previous studies suggest that they can enhance the tumor uptake and retention of radiopharmaceuticals, resulting in improved therapeutic efficacy (*18*–*23*). Further clinical translation studies also validate the promise of this strategy to be a platform technology for radiopharmaceutical development (*24*–*26*). Therefore, we were curious whether attaching an albumin-binder moiety to the FAPI molecules would improve the FAP-targeted radiotherapy efficacy at the expense of increased retention in blood.

In this study, 2 albumin binder–FAPI conjugates, TEFAPI-06 and TEFAPI-07, were developed by logistic fabrication of 3 functional components: a quinoline-based FAPI originating from FAPI-04, a chelator (i.e., DOTA group) that allows radionuclide labeling for imaging (^68^Ga or ^86^Y) or therapy (^177^Lu), and an albumin binder: 4-(*p*-iodophenyl) butyric acid moiety (TEFAPI-06) or truncated Evans blue moiety (TEFAPI-07). The purpose of the study was to evaluate whether the modification improves tumor retention in vivo and which albumin binder better matches FAPI molecules. A series of detailed experiments and comparisons, including cell binding assays, a PET imaging study, a biodistribution study, and a radiotherapy study, was performed. The results demonstrated these 2 albumin binder–conjugated FAPI radiotracers to have high FAP binding affinity and specificity, enhanced tumor retention, and improved radiotherapy efficacy.

## MATERIALS AND METHODS

### Ligands and Radionuclides

The synthesis route and chemical characterization of TEFAPI-06 and TEFAPI-07 are described in Supplemental Figures 1–20 (supplemental materials are available at http://jnm.snmjournals.org). ^68^Ga-Cl_3_ was eluted with a solution of 0.6 M hydrochloride from a ^68^Ge–^68^Ga generator (iThemba LABS). ^86^Y-Cl_3_ was produced with a 14.6-MeV cyclotron; the target design follows our previous report (*27*), and the purification procedure follows the previous protocol (*28*). ^177^Lu-Cl_3_ in a solution of 0.1 M hydrochloride was purchased from ITG.

### Radiolabeling and Stability In Vitro

The radiolabeling of ^68^Ga, ^86^Y, and ^177^Lu was performed by incubation with 50 nmol of precursor at pH 4.5–5.0 at 90°C for 10 min. The product was purified by C18 column extraction, and the radiochemical purity was determined by high-performance liquid chromatography equipped with a radioactivity detector. The stability of ^177^Lu-TEFAPI-06 and ^177^Lu-TEFAPI-07 in saline and human serum was monitored from 2 to 168 h using radio–high-performance liquid chromatography (*13*). More details about the radiochemistry, the quality control testing, and the stability assay can be found in the supplemental materials (section 3).

### Cell Culture and Assay

The human fibrosarcoma cell line (HT-1080), and the HT-1080 cell line transfected with human FAP gene (HT-1080-FAP, from WuXi AppTec), were cultivated in Eagle minimum essential medium containing 10% fetal bovine serum, 1% antibiotic–antimycotic, and a 4 μg/mL concentration of blasticidin S at 37°C under conditions of 5% carbon dioxide. For competition assays, HT-1080-FAP cells were seeded in 6-well plates and cultivated until they reached about 1.2 × 10^6^ cells per well. The cells were incubated simultaneously with unlabeled FAPI-04, TEFAPI-06, or TEFAPI-07 (10^−5^–10^−9^ M) with ^68^Ga-FAPI-04 in 1 mL of fresh medium without fetal bovine serum for 1 h. The medium was removed, and the cells were washed twice with phosphate-buffered saline (PBS). Subsequently, the cells were lysed with 0.5 mL of 1 M NaOH and washed with 0.5 mL of PBS twice, and the NaOH (0.5 mL) and PBS (0.5 mL × 2) were collected to determine the uptake counts. For saturation binding assays, HT-1080-FAP and HT-1080 cells were seeded in 24-well plates and cultivated until they reached about 2 × 10^5^ cells per well. ^68^Ga-FAPI-04, ^68^Ga-TEFAPI-06, or ^68^Ga-TEFAPI-07 was diluted to a concentration 0.01–200 nM in fresh medium without fetal bovine serum. The cells were incubated in the above solution for 1 h and then washed twice with PBS. The lysed cells and the PBS for washing were collected to determine the counts.

### Tumor-Bearing Animal Models

All animal care and experimental procedures were performed by following the animal protocols (CCME-LiuZB-2) approved by the ethics committee of Peking University. The mice were from the Beijing Vital River Laboratory Animal Technology Co., Ltd. For cell-line–derived xenograft models, 5 × 10^6^ HT-1080-FAP or HT-1080 cells were subcutaneously inoculated into the right shoulder of 6-wk-old female nu/nu-mice. To establish the patient-derived xenograft (PDX) model, tumor specimens were obtained from patients who underwent presurgical ^68^Ga-FAPI-04 PET/CT imaging to confirm that the tumor was FAP-positive. After surgical resection, the tumor specimens were immediately placed in ready-to-use fresh tissue preservation solution (TM2701-100) and transported under refrigerated conditions within 2 h. The research protocol was approved by the Institutional Ethics Committee of Peking Union Medical College Hospital (JS-2628). Six-week-old female nonobese diabetic/severe combined immunodeficiency (NOD/SCID) mice were used to establish the PDX models. After being removed from the preservation solution, the tumor specimens were immediately immersed in sterile PBS solution and minced with scissors, and the fragments were then implanted subcutaneously into the left and right shoulders of the mice, which were anesthetized with isoflurane in advance. Engraftment efficiency was determined by ^68^Ga-FAPI-04 PET/CT imaging (Supplemental Fig. 21A). Immunohistochemical staining (Supplemental Fig. 21B) demonstrated that the pancreatic cancer PDX model used in this study was indeed FAP-overexpressed.

### Small-Animal PET Imaging

All PET scans were performed on a Mediso nanoScan PET 122S small-animal PET/CT imaging system. For the 60-min dynamic PET scan, 29.6–37.0 MBq of ^68^Ga-TEFAPI-06 or ^68^Ga-TEFAPI-07 were given to healthy NOD/SCID mice through tail-vein injection. The static PET imaging was performed on mice bearing pancreatic PDX tumors, HT-1080-FAP tumors, and HT-1080 tumors at the indicated time points after intravenous injection of 7.4–11.1 MBq of ^86^Y-FAPI-04,^86^Y-TEFAPI-06, or ^86^Y-TEFAPI-07.

### Small-Animal SPECT Imaging

SPECT scans were performed on a Mediso nanoSPECT/CT imaging system. ^177^Lu-TEFAPI-06 or ^177^Lu-TEFAPI-07 SPECT imaging was performed on pancreatic cancer PDX–bearing mice at the indicated time points after intravenous injection of 37 MBq of ^177^Lu-TEFAPI-06 or ^177^Lu-TEFAPI-07, respectively.

### Biodistribution Study

PDX-bearing mice were injected with 925.0 kBq of ^177^Lu-TEFAPI-06 or ^177^Lu-TEFAPI-07 for an ex vivo biodistribution study. The mice were killed at 24 h and 96 h after injection, the counts of the different organs were measured with a γ-counter, and the data were normalized to percentage injected dose (%ID)/g using 1% of total counts.

### Radiotherapy Study

^68^Ga-FAPI-04 PET imaging was performed to evaluate the tumor volume, and the mice were treated when their average tumor volume reached 35 mm^3^. The PDX-bearing mice (6 groups, 7–9 mice per group) were treated by saline, 3.7 MBq of ^177^Lu-FAPI-04, 1.85 MBq of ^177^Lu-TEFAPI-06, 3.7 MBq of ^177^Lu-TEFAPI-06, 1.85 MBq of ^177^Lu-TEFAPI-07, or 1.85 MBq of ^177^Lu-TEFAPI-07. Tumor volume and body weight were monitored every 2 or 3 d, and the animals were euthanized when the tumor volume exceeded 1,000 mm^3^. Histopathologic staining was performed with an antihuman FAP monoclonal antibody (ab207178; Abcam), and hematoxylin and eosin staining was performed as previously described (*29*).

## RESULTS

### Radiochemistry and Stability In Vitro

**FIGURE 1.**
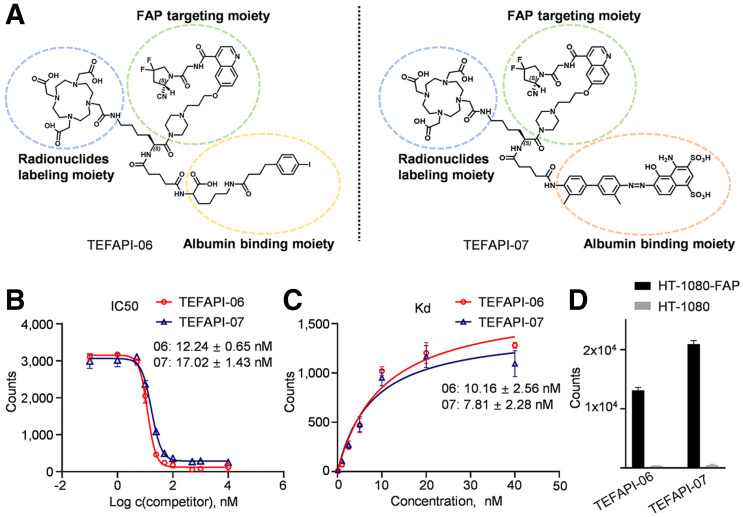
(A) Chemical structures of TEFAPI-06 and TEFAPI-07. (B) Competition assays of TEFAPI-06 and TEFAPI-07. (C) Saturation binding assays of radiolabeled TEFAPI-06 and TEFAPI-07. (D) Cellular uptake assays of ^68^Ga-TEFAPI-06 and ^68^Ga-TEFAPI-07 in HT-1080-FAP and HT-1080 cells. IC_50_ = half-maximal inhibitory concentration.

The radiolabeling yield of TEFAPI-06 and TEFAPI-07 (Fig. 1A) was over 90%, and the radiochemical purity was over 99% (*n* > 20). The specific activity of ^68^Ga-FAPI-04, ^68^Ga-TEFAPI-06, and ^68^Ga-TEFAPI-07 was 5.2–6.7 GBq/μmol. The specific activity of ^86^Y-TEFAPI-06 and ^86^Y-TEFAPI-07 was 2.2–3.4 GBq/μmol. The specific activity of ^177^Lu-FAPI-04, ^177^Lu-TEFAPI-06, and ^177^Lu-TEFAPI-07 was 2.9–4.4 GBq/μmol. Stability of ^177^Lu-TEFAPI-06 and ^177^Lu-TEFAPI-07 in saline and human serum was analyzed using radio–high-performance liquid chromatography, as shown in Supplemental Figure 22. The radiochemistry purity of both ^177^Lu-TEFAPI-06 and ^177^Lu-TEFAPI-07 was still over 90% after incubation in saline and human serum for 7 d.

### Binding Assay

As shown in Figure 1B and Supplemental Figure 23A, cellular uptake of ^68^Ga-FAPI-04 can be significantly inhibited by treatment with cold TEFAPI-06 and TEFAPI-07. The ligand concentrations required for 50% inhibition (half-maximal inhibitory concentration) of TEFAPI-06 and TEFAPI-07 are 12.24 ± 0.65 nM and 17.02 ± 1.43 nM, respectively. The dissociation constants of ^68^Ga-TEFAPI-06 and ^68^Ga-TEFAPI-07 were 10.16 ± 2.56 nM and 7.81 ± 2.28 nM (Fig. 1C), respectively, which are comparable to that of ^68^Ga-FAPI-04 (1.91 ± 0.62 nM, Supplemental Fig. 23B). As shown in Figure 1D, both ^68^Ga-TEFAPI-06 and ^68^Ga-TEFAPI-07 exhibited almost negligible uptake in HT-1080 cells but had significant uptake in HT-1080-FAP cells. We also performed the binding assays in 0.05% human serum albumin (*20*), with the following results. The half-maximal inhibitory concentrations of TEFAPI-06 and TEFAPI-07 were 11.39 ± 1.15 nM and 27.68 ± 5.00 nM, respectively, in the presence of albumin. The dissociation constants of TEFAPI-06 and TEFAPI-07 were 4.37 ± 0.81 nM and 19.12 ± 5.54, respectively, in the absence of albumin. The half-maximal inhibitory concentration and dissociation constant of TEFAPI-07 were slightly impacted by the presence of albumin, which may be the reason why blood clearance was faster than for TEFAPI-06.

### Small-Animal PET Imaging

**FIGURE 2.**
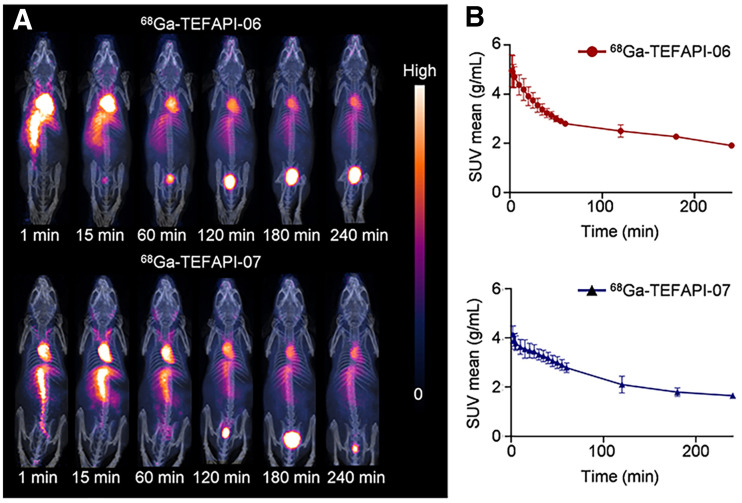
(A) Dynamic PET imaging of ^68^Ga-TEFAPI-06 and ^68^Ga-TEFAPI-07 in healthy NOD/SCID mice. (B) Corresponding blood time–activity curves of ^68^Ga-TEFAPI-06 and ^68^Ga-TEFAPI-07.

To evaluate the in vivo pharmacokinetics of these 2 radiotracers, dynamic PET imaging of ^68^Ga-TEFAPI-06 and ^68^Ga-TEFAPI-07 was performed on healthy NOD-SCID mice. The signal in heart peaked rapidly at about 2 min after injection and then declined gradually. For ^68^Ga-TEFAPI-06, the signal decreased by 35.70% ± 4.74% from 10 to 60 min after injection, a decrease that was greater than that of ^68^Ga-TEFAPI-07 (23.15% ± 2.16%), whereas from 60 to 240 min after injection, the signal decreased by 31.80% ± 1.15% and 40.56% ± 5.25% for ^68^Ga-TEFAPI-06 and ^68^Ga-TEFAPI-07, respectively, resulting in a similar proportion in the decrease of these 2 radiotracers from 10 to 240 min, at 56.18% ± 2.50% and 54.28% ± 4.98% for ^68^Ga-TEFAPI-06 and ^68^Ga-TEFAPI-07, respectively. As shown in Figure 2, for both ^68^Ga-TEFAPI-06 and ^68^Ga-TEFAPI-07, most of the radioactivity was retained in the blood circulation during the monitoring period, and the uptake in other organs, such as the liver, spleen, and kidney, was lower than in the heart or main blood vessels.

**FIGURE 3.**
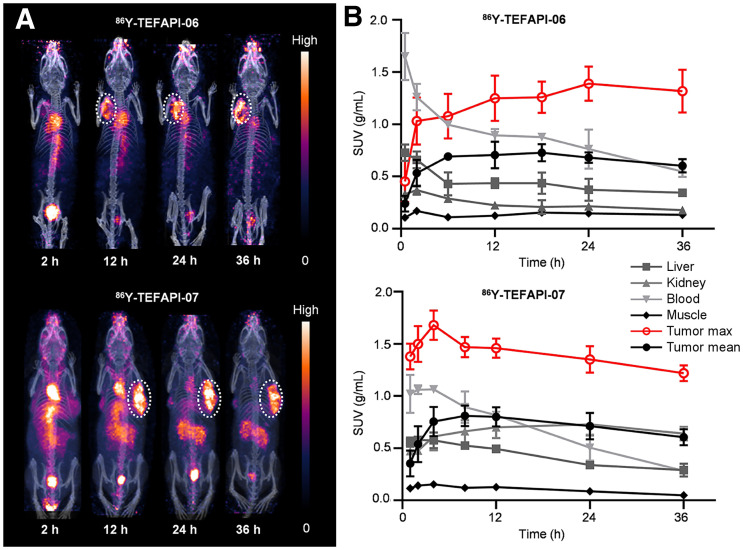
(A) PET imaging of ^86^Y-TEFAPI-06 and ^86^Y-TEFAPI-07 in PDX-bearing mice. (B) Time–activity curves for tumor and major organs of ^86^Y-TEFAPI-06 and ^86^Y-TEFAPI-07.

To identify the tumor-targeting ability and monitor the in vivo pharmacokinetics quantitatively over a longer period, TEFAPI-06, TEFAPI-07, and FAPI-04 were labeled with the radionuclide ^86^Y, which has a half-life of 14.7 h, and the PET imaging was performed using pancreatic cancer PDX–bearing mice. As shown in Figure 3 and Supplemental Figure 24, for both ^86^Y-TEFAPI-06 and ^86^Y-TEFAPI-07, tumor was completely visible at 2 h after injection. The tumor SUV_mean_ of ^86^Y-TEFAPI-06 peaked at 0.73 at 18 h after injection, and that of ^86^Y-TEFAPI-07 peaked at 0.81 at 8 h after injection. Then, the tumor SUV_mean_ decreased slowly but still remained high until 36 h after injection, with a value of 0.602 and 0.606 for ^86^Y-TEFAPI-06 and ^86^Y-TEFAPI-07, respectively. However, the tumor SUV_mean_ of ^86^Y-FAPI-04 peaked at 0.35 at 0.2 h after injection and then decreased rapidly, and the areas under the curve for TEFAPI-07 and TEFAPI-06 were 35.5-fold and 37.9-fold that for FAPI-04.

**FIGURE 4.**
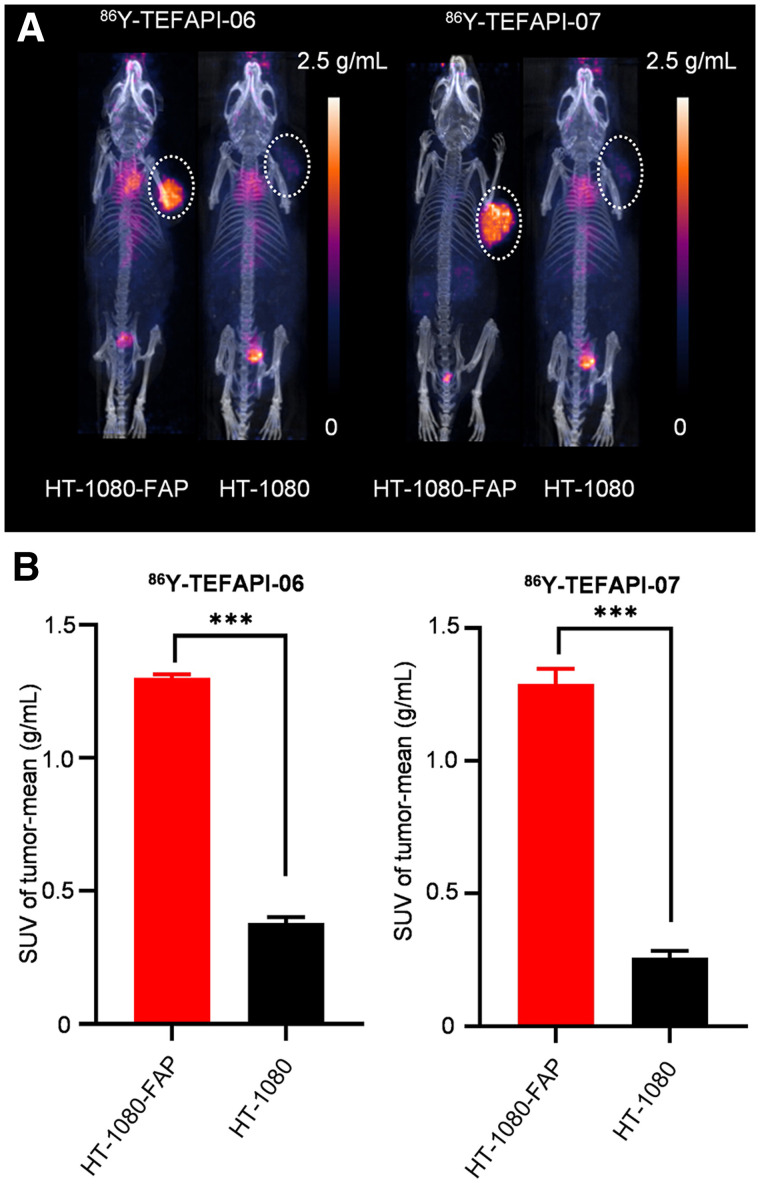
(A) PET imaging of ^86^Y-TEFAPI-06 and ^86^Y-TEFAPI-07 in HT-1080-FAP and HT-1080 tumor–bearing mice at 24 h after injection. (B) Uptake of ^86^Y-TEFAPI-06 and ^86^Y-TEFAPI-07 in HT-1080-FAP and HT-1080 tumors at 24 h after injection. ***P < 0.001.

To further confirm the FAP specificity in vivo of these 2 radiotracers, PET imaging of HT-1080-FAP and HT-1080 tumor–bearing mice was performed. As shown in Figure 4, Supplemental Figure 25, and Supplemental Figure 26, the uptake of ^86^Y-TEFAPI-06 and ^86^Y -TEFAPI-07 in HT-1080-FAP tumors was consistently 2- to 6-fold higher than that in HT-1080 tumors. A blocking study was also performed, as shown in Supplemental Figure 27; tumor uptake decreased at 12 h and 24 h after treatment with cold TEFAPI-06 and TEFAPI-07.

### Small-Animal SPECT Imaging

To further characterize these 2 molecules, SPECT imaging was conducted on PDX tumor models for a longer time. As shown in Supplemental Figure 28, high tumor–to–nontargeted-tissue signal ratios were observed for both ^177^Lu-TEFAPI-06 and ^177^Lu-TEFAPI-07 until 144 h after injection. The blood circulation properties of these 2 molecules were similar to those found in the previous PET study.

### Biodistribution Study

**FIGURE 5.**
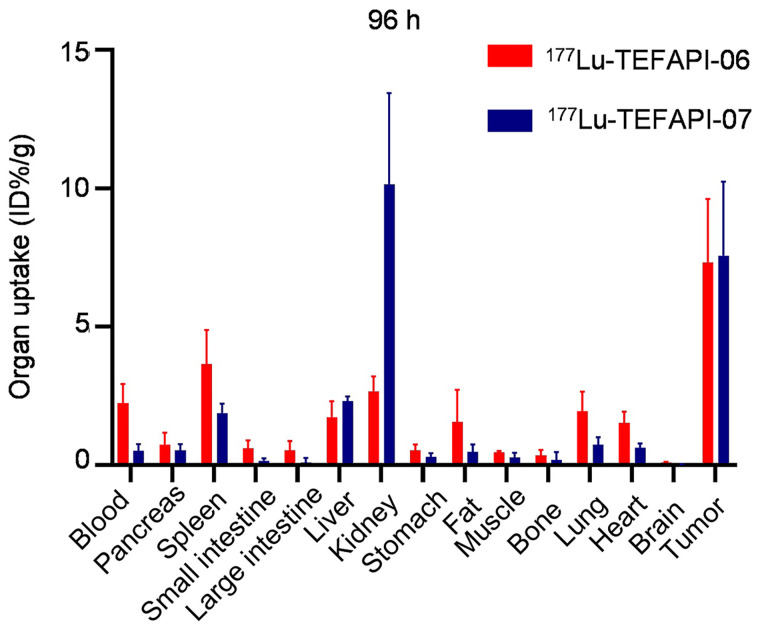
Biodistribution study of ^177^Lu-TEFAPI-06 and ^177^Lu-TEFAPI-07 in pancreatic cancer PDX–bearing mice at 96 h after injection (*n* = 5).

To further evaluate the metabolic properties in vivo, biodistribution studies using the pancreatic cancer PDX–bearing mice were performed. As shown in Figure 5 and Table 1, the tumor uptake of ^177^Lu-TEFAPI-06 and ^177^Lu-TEFAPI-07 were, respectively, 8.68 ± 0.73 %ID/g and 7.87 ± 2.08 %ID/g at 24 h after injection, and the tumor-to-liver ratios were 2.91 and 2.45, respectively. The tumor uptake remained high until 96 h after injection, at 7.33 ± 2.28 %ID/g and 7.57 ± 2.68 %ID/g for ^177^Lu-TEFAPI-06 and ^177^Lu-TEFAPI-07, respectively, and the tumor-to-liver ratios increased to 4.21 and 3.28, respectively. As is consistent with the results of PET and SPECT imaging, the kidney uptake of ^177^Lu-TEFAPI-07 remained high at both 24 and 96 h after injection, at 8.67 ± 2.30 and 10.16 ± 3.28 %ID/g, respectively, whereas the kidney uptake of ^177^Lu-TEFAPI-06 was much lower, at 2.66 ± 0.54 %ID/g, at 96 h after injection. By comparison, the blood clearance of ^177^Lu-TEFAPI-06 (24 h, 13.32 ± 1.33 %ID/g; 96 h, 2.25 ± 0.68 %ID/g) was slower than that of ^177^Lu-TEFAPI-07 (24 h, 5.64 ± 1.50 %ID/g; 96 h, 0.51 ± 0.24 %ID/g).

**TABLE 1.** Biodistribution Results for ^177^Lu-TEFAPI-06 and ^177^Lu-TEFAPI-07

	^177^Lu-TEFAPI-06				^177^Lu-TEFAPI-07			
	24 h		96 h		24 h		96 h	
Organ	Mean	SD	Mean	SD	Mean	SD	Mean	SD
Blood	12.32	1.33	2.25	0.68	5.64	1.49	0.51	0.24
Pancreas	2.01	0.26	0.75	0.42	2.16	0.69	0.53	0.22
Spleen	3.00	0.66	3.66	1.22	2.69	0.87	1.88	0.34
Small intestine	2.55	0.64	0.61	0.28	1.19	0.26	0.16	0.09
Large intestine	1.66	0.27	0.53	0.35	1.15	0.33	0.10	0.15
Liver	2.98	0.64	1.74	0.57	3.21	0.81	2.31	0.17
Kidney	3.20	0.84	2.66	0.54	8.67	2.30	10.16	3.28
Stomach	1.75	0.17	0.54	0.21	1.20	0.45	0.29	0.14
Fat	1.86	0.90	1.55	1.17	1.82	1.18	0.48	0.25
Muscle	1.09	0.18	0.45	0.05	1.00	0.37	0.28	0.16
Bone	1.32	0.30	0.35	0.20	1.71	0.63	0.18	0.28
Lung	4.33	1.44	1.95	0.70	2.58	0.75	0.73	0.28
Heart	3.70	1.59	1.51	0.42	2.53	1.19	0.62	0.17
Brain	0.37	0.08	0.09	0.03	0.23	0.09	−0.01	0.04
Tumor	8.68	0.73	7.33	2.28	7.87	2.08	7.57	2.68

Data are %ID/g.

### Radiotherapy Study

**FIGURE 6.**
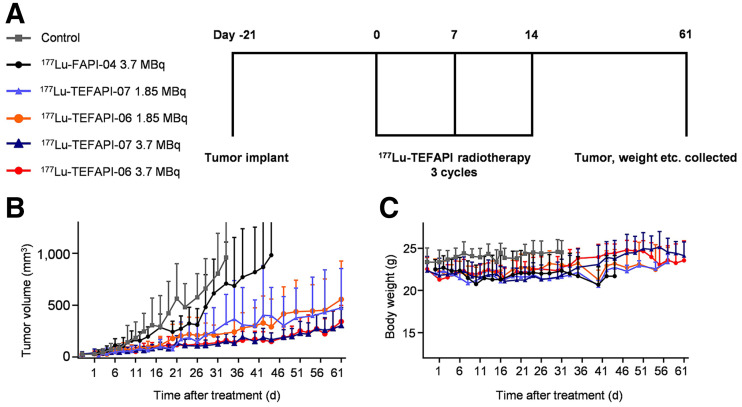
Treatment assay of ^177^Lu-TEFAPI-06 and ^177^Lu-TEFAPI-07 in pancreatic cancer PDX-bearing mice. (A) Design of therapy protocols and treatment scheme. (B) Tumor growth curve after treatment (*n* = 7–9 mice for each group). (C) Body weight change after treatment.

To make the assessment of therapeutic efficacy more relevant to the clinical setting, pancreatic cancer PDX–bearing mice were used for the indicated radiotherapy study (Fig. 6A). In a comparison to the group treated by saline or 3.7 MBq of ^177^Lu-FAPI-04, the groups treated with 1.85 MBq or 3.7 MBq of ^177^Lu-TEFAPI-06 and ^177^Lu-TEFAPI-07, respectively, showed remarkable suppression of tumor growth (Fig. 6B). No statistical difference in treatment efficacy was observed between ^177^Lu-TEFAPI-06 and ^177^Lu-TEFAPI-07. This result corroborates the PET imaging and biodistribution studies, as they showed equally high uptake in the tumors. Except for the control group treated with only saline, transient weight loss was observed for all treatment groups, including ^177^Lu-FAPI-04, but then returned to the healthy level 7 d after the initial treatment. Hematoxylin and eosin staining of the main organs revealed that side effects from ^177^Lu-TEFAPI-06 and ^177^Lu-TEFAPI-07 treatment were almost negligible (Supplemental Fig. 29).

## DISCUSSION

The purpose of this study was to develop FAPI-based radiopharmaceuticals that are more effective than the existing candidates for FAP-targeted radiotherapy. Two different albumin binders, 4-(*p*-iodophenyl) butyric acid and truncated Evans blue moieties, were chosen to be attached with FAPI-04. The resulting TEFAPI-06 and TEFAPI-07 were synthesized and radiolabeled with ^68^Ga, ^86^Y, and ^177^Lu. The radiolabeled TEFAPIs exhibited good stability in saline and human serum and high FAP binding affinity in vitro. In addition, SPECT imaging and biodistribution studies of ^177^Lu-TEFAPI-06 and ^177^Lu-TEFAPI-07 showed that tumor uptake was still notable even at 6 d after the injection. Meanwhile, almost no radioactive signal could be detected for ^177^Lu-FAPI-04 at 24 h after injection. We also wondered whether further modifications of the structure may lengthen the blood circulation and, thus, increase the tumor accumulation. However, it can be challenging to balance treatment efficacy against potential side effects from blood circulation.

With regard to the clearance pathway, there was no significant difference in uptake between ^177^Lu-TEFAPI-06 and ^177^Lu-TEFAPI-07 in tumor and main organs, except for the kidney. For TEFAPI-07, both the PET and the SPECT imaging results showed significantly higher kidney uptake than that of TEFAPI-06. Of note, imaging indicated that there was no obvious clearance of TEFAPI-07 from the kidneys over time, a finding that was consistent with the results of the biodistribution study. Besides, because both TEFAPI-06 and ^177^Lu-TEFAPI-07 have relatively longer blood circulation than the classic radiopharmaceuticals, the side effects may not be negligible. Therefore, a comprehensive hematoxylin- and eosin-staining study of major organs was performed, and no tissue damage was observed (Supplemental Fig. 29).

As reported in previous studies, the radiolabeled albumin binder may target the tumor because of enhanced permeability and retention of albumin (*30**,**31*). Thus, we were curious about whether the enhanced tumor uptake and retention of ^177^Lu-TEFAPI-06 and ^177^Lu-TEFAPI-07 are FAP-dependent. The PET imaging results of FAP-positive (HT-1080-FAP) and FAP-negative (HT-1080) tumor–bearing mice showed much higher uptake by FAP-positive tumors than by FAP-negative tumors, demonstrating that the higher tumor uptake was dependent on the FAP-targeting ability in vivo. For the blocking study, the tumor uptake of ^68^Ga-FAPI-04 decreased significantly when the mice were treated with cold TEFAPI-06 and TEFAPI-07 until 24 h after injection—a finding that supported the possibility that the prolonged tumor retention of these 2 radiotracers was also dependent mainly on their excellent FAP-targeting ability in vivo.

## CONCLUSION

In this study, 2 albumin binder–conjugated FAPIs, denoted as TEFAPI-06 and TEFAPI-07, were developed to optimize the pharmacokinetics of current FAPI radiopharmaceuticals for cancer radiotherapy. Compared with ^177^Lu-FAPI-04, both ^177^Lu-TEFAPI-06 and ^177^Lu-TEFAPI-07 showed enhanced uptake and retention in tumors. The tumor accumulations were highly FAP-selective and resulted in remarkable inhibition of PDX tumor growth, with negligible side effects. Their promising pharmacokinetics warrant further investigations toward clinical translation for the treatment of FAP-positive cancers.

## DISCLOSURE

This work was funded by the Beijing Municipal Natural Science Foundation (grant Z200018), the Special Foundation of the Beijing Municipal Education Commission (grant 3500-12020123), the National Natural Science Foundation of China (grants U1867209 and 21778003), the Ministry of Science and Technology of the People’s Republic of China (grant 2017YFA0506300), and the Li Ge-Zhao Ning Life Science Youth Research Foundation (grant LGZNQN202004). Mengxin Xu, Pu Zhang, Junyi Chen, and Zhibo Liu are consultants for Borui Biotech. No other potential conflict of interest relevant to this article was reported.

## Notes

KEY POINTS **QUESTION:** Do albumin binder–conjugated FAPI radiopharmaceuticals have high FAP specificity and provide enhanced tumor retention and improved radiotherapy efficiency? **PERTINENT FINDINGS:** Albumin binder–conjugated FAPI radiopharmaceuticals exhibited notably improved tumor uptake and retention compared with the original FAPI tracers and showed remarkable inhibition of PDX tumor growth, with negligible side effects. **IMPLICATIONS FOR PATIENT CARE:** Albumin binder conjugation is a promising strategy in the development of FAP-targeted radiopharmaceuticals for treating cancers.

## References

[1] Hamson EJ Keane FM Tholen S Schilling O Gorrell MD. Understanding fibroblast activation protein (FAP): substrates, activities, expression and targeting for cancer therapy. Proteomics Clin Appl. 2014;8:454–463. 24470260 10.1002/prca.201300095

[2] Park JE Lenter MC Zimmermann RN Garin-Chesa P Old LJ Rettig WJ. Fibroblast activation protein, a dual specificity serine protease expressed in reactive human tumor stromal fibroblasts. J Biol Chem. 1999;274:36505–36512. 10593948 10.1074/jbc.274.51.36505

[3] Li H Fan X Houghton J. Tumor microenvironment: the role of the tumor stroma in cancer. J Cell Biochem. 2007;101:805–815. 17226777 10.1002/jcb.21159

[4] Calais J. FAP: the next billion dollar nuclear theranostics target? J Nucl Med. 2020;61:163–165. 31924719 10.2967/jnumed.119.241232

[5] Lo A Wang L-CS Scholler J. Tumor-promoting desmoplasia is disrupted by depleting FAP-expressing stromal cells. Cancer Res. 2015;75:2800–2810. 25979873 10.1158/0008-5472.CAN-14-3041 PMC4506263

[6] Fischer E Chaitanya K Wüest T. Radioimmunotherapy of fibroblast activation protein positive tumors by rapidly internalizing antibodies. Clin Cancer Res. 2012;18:6208–6218. 22992515 10.1158/1078-0432.CCR-12-0644

[7] Jansen K Heirbaut L Verkerk R. Extended structure–activity relationship and pharmacokinetic investigation of (4-quinolinoyl)glycyl-2-cyanopyrrolidine inhibitors of fibroblast activation protein (FAP). J Med Chem. 2014;57:3053–3074. 24617858 10.1021/jm500031w

[8] Loktev A Lindner T Mier W. A tumor-imaging method targeting cancer-associated fibroblasts. J Nucl Med. 2018;59:1423–1429. 29626120 10.2967/jnumed.118.210435 PMC6126438

[9] Lindner T Altmann A Krämer S. Design and development of ^99m^Tc-labeled FAPI tracers for SPECT imaging and ^188^Re therapy. J Nucl Med. 2020;61:1507–1513. 32169911 10.2967/jnumed.119.239731 PMC7539653

[10] Giesel FL Adeberg S Syed M. FAPI-74 PET/CT using either ^18^F-AlF or cold-kit ^68^Ga labeling: biodistribution, radiation dosimetry, and tumor delineation in lung cancer patients. J Nucl Med. 2021;62:201–207. 32591493 10.2967/jnumed.120.245084 PMC8679591

[11] Kratochwil C Flechsig P Lindner T. ^68^Ga-FAPI PET/CT: tracer uptake in 28 different kinds of cancer. J Nucl Med. 2019;60:801–805. 30954939 10.2967/jnumed.119.227967 PMC6581228

[12] Windisch P Zwahlen DR Koerber SA. Clinical results of fibroblast activation protein (FAP) specific PET and implications for radiotherapy planning: systematic review. Cancers (Basel). 2020;12:2629. 10.3390/cancers12092629 PMC7564725 32942573

[13] Lindner T Loktev A Altmann A. Development of quinoline-based theranostic ligands for the targeting of fibroblast activation protein. J Nucl Med. 2018;59:1415–1422. 29626119 10.2967/jnumed.118.210443

[14] Loktev A Lindner T Burger E-M. Development of fibroblast activation protein–targeted radiotracers with improved tumor retention. J Nucl Med. 2019;60:1421–1429. 30850501 10.2967/jnumed.118.224469 PMC6785792

[15] Watabe T Liu Y Kaneda-Nakashima K. Theranostics targeting fibroblast activation protein in the tumor stroma: ^64^Cu- and ^225^Ac-labeled FAPI-04 in pancreatic cancer xenograft mouse models. J Nucl Med. 2020;61:563–569. 31586001 10.2967/jnumed.119.233122 PMC7198371

[16] Ballal S Yadav MP Kramer V. A theranostic approach of [^68^Ga]Ga-DOTA.SA.FAPi PET/CT-guided [^177^Lu]Lu-DOTA.SA.FAPi radionuclide therapy in an end-stage breast cancer patient: new frontier in targeted radionuclide therapy. Eur J Nucl Med Mol Imaging. 2021;48:942–944. 32783111 10.1007/s00259-020-04990-w

[17] Liu Z Chen X. Simple bioconjugate chemistry serves great clinical advances: albumin as a versatile platform for diagnosis and precision therapy. Chem Soc Rev. 2016;45:1432–1456. 26771036 10.1039/c5cs00158g PMC5227548

[18] Müller C Farkas R Borgna F Schmid RM Benešová M Schibli R. Synthesis, radiolabeling, and characterization of plasma protein-binding ligands: potential tools for modulation of the pharmacokinetic properties of (radio)pharmaceuticals. Bioconjug Chem. 2017;28:2372–2383. 28898054 10.1021/acs.bioconjchem.7b00378

[19] Benešová M Umbricht CA Schibli R Müller C. Albumin-binding PSMA ligands: optimization of the tissue distribution profile. Mol Pharm. 2018;15:934–946. 29400475 10.1021/acs.molpharmaceut.7b00877

[20] Kelly JM Jeitner TM Ponnala S. A trifunctional theranostic ligand targeting fibroblast activation protein-α (FAPα). Mol Imaging Biol. 2021;23:686–696. 33721173 10.1007/s11307-021-01593-1

[21] Kelly JM Amor-Coarasa A Nikolopoulou A. Dual-target binding ligands with modulated pharmacokinetics for endoradiotherapy of prostate cancer. J Nucl Med. 2017;58:1442–1449. 28450562 10.2967/jnumed.116.188722

[22] Lau J Jacobson O Niu G Lin KS Bénard F Chen X. Bench to bedside: albumin binders for improved cancer radioligand therapies. Bioconjug Chem. 2019;30:487–502. 30616340 10.1021/acs.bioconjchem.8b00919

[23] Kuo HT Lin KS Zhang Z. Novel ^177^Lu-labeled albumin-binder-conjugated PSMA-targeting agents with extremely high tumor uptake and enhanced tumor-to-kidney absorbed dose ratio. J Nucl Med. 2021;62:521–527. 32859704 10.2967/jnumed.120.250738 PMC8049373

[24] Kramer V Fernández R Lehnert W. Biodistribution and dosimetry of a single dose of albumin-binding ligand [^177^Lu]Lu-PSMA-ALB-56 in patients with mCRPC. Eur J Nucl Med Mol Imaging. 2021;48:893–903. 32949253 10.1007/s00259-020-05022-3 PMC8036212

[25] Zhang J Hao W Weiss OJ Cheng Y Chen X. Safety, pharmacokinetics and dosimetry of a long-acting radiolabeled somatostatin analogue ^177^Lu-DOTA-EB-TATE in patients with advanced metastatic neuroendocrine tumors. J Nucl Med. 2018;59:1699–1705. 29653971 10.2967/jnumed.118.209841 PMC6225536

[26] Wang H Cheng Y Zhang J Jie Z Chen X. Response to single low-dose ^177^Lu-DOTA-EB-TATE treatment in patients with advanced neuroendocrine neoplasm: a prospective pilot study. Theranostics. 2018;8:3308–3316. 29930731 10.7150/thno.25919 PMC6010978

[27] Wang Q Wang Y Ding J. A bioorthogonal system reveals antitumour immune function of pyroptosis. Nature. 2020;579:421–426. 32188939 10.1038/s41586-020-2079-1

[28] Avila-Rodriguez MA Nye JA Nickles RJ. Production and separation of non-carrier-added ^86^Y from enriched ^86^Sr targets. Appl Radiat Isot. 2008;66:9–13. 17869530 10.1016/j.apradiso.2007.07.027

[29] Ren J Xu M Chen J. PET imaging facilitates antibody screening for synergistic radioimmunotherapy with a ^177^Lu-labeled αPD-L1 antibody. Theranostics. 2021;11:304–315. 33391476 10.7150/thno.45540 PMC7681088

[30] Kelly J Amor-Coarasa A Ponnala S. Trifunctional PSMA-targeting constructs for prostate cancer with unprecedented localization to LNCaP tumors. Eur J Nucl Med Mol Imaging. 2018;45:1841–1851. 29623376 10.1007/s00259-018-4004-5

[31] Kelly JM Amor-Coarasa A Ponnala S. Albumin-binding PSMA ligands: implications for expanding the therapeutic window. J Nucl Med. 2019;60:656–663. 30552199 10.2967/jnumed.118.221150

